# Genomic Sequencing of *Phyllosticta citriasiana* Provides Insight Into Its Conservation and Diversification With Two Closely Related *Phyllosticta* Species Associated With Citrus

**DOI:** 10.3389/fmicb.2019.02979

**Published:** 2020-01-10

**Authors:** Mingshuang Wang, Bei Liu, Ruoxin Ruan, Yibing Zeng, Jinshui Luo, Hongye Li

**Affiliations:** ^1^College of Life and Environmental Sciences, Hangzhou Normal University, Hangzhou, China; ^2^Key Laboratory of Molecular Biology of Crop Pathogens and Insects, Ministry of Agriculture, Institute of Biotechnology, Zhejiang University, Hangzhou, China; ^3^Hangzhou Academy of Agricultural Sciences, Hangzhou, China; ^4^Fujian Institute of Tropical Crops, Zhangzhou, China

**Keywords:** *Phyllosticta citriasiana*, pomelo tan spot, comparative genomics, secreted proteins, secondary metabolism

## Abstract

*Phyllosticta capitalensis*, *Phyllosticta citricarpa*, and *Phyllosticta citriasiana* are three very important *Phyllosticta* species associated with citrus. *P. capitalensis* is an endophyte fungus of citrus while *P. citricarpa* can cause black spot of citrus (e.g., oranges and mandarins). *P. citriasiana* was identified recently which is the causal agent of the pomelo tan spot. Here, we present the ∼34 Mb genome of *P. citriasiana*. The genome is organized in 92 contigs, encompassing 9202 predicted genes. Comparative genomic analyses with two other *Phyllosticta* species (*P. citricarpa* and *P. capitalensis*) associated with citrus was conducted to understand their evolutionary conservation and diversification. Pair-wise genome alignments revealed that these species are highly syntenic. All species encode similar numbers of CAZymes and secreted proteins. However, the molecular functions of the secretome showed that each species contains some enzymes with distinct activities. The three *Phyllosticta* species investigated shared a core set of 7261 protein families. *P. capitalensis* had the largest set of orphan genes (1991), in complete contrast to that of *P. citriasiana* (364) and *P. citricarpa* (262). Most of the orphan genes are functionally unknown, but they contain a certain number of species-specific secreted proteins. A total of 23 secondary metabolites biosynthesis clusters were identified in the three *Phyllosticta* species, 21 of them being highly conserved among these species while the remaining two showed whole cluster gain and loss polymorphisms or gene content polymorphisms. Taken together, our study reveals insights into the genetic mechanisms of host adaptation of three species of *Phyllosticta* associated with citrus and paves the way to identify effectors that function in infection of citrus plants.

## Introduction

Citrus Black Spot (CBS), caused by *Phyllosticta citricarpa*, is an important disease of citrus, it can affect almost all grown citrus cultivars, including sweet orange (*Citrus sinensis*), mandarin (*C. reticulata* and *C. unshiu*), lime (*C. aurantium*) and lemon (*C. limon*) (Wang et al., 2012; Guarnaccia et al., 2019). This disease mainly causes black lesions in the fruits, making the fruits unsuitable for the fresh market. When the disease is severe, yield losses are significant due to premature fruit drop (Kotzé, 1981). CBS mainly occurs in humid subtropical regions, including Asia, Africa, South America, Australia, and most recently, Florida (Kotzé, 1981; Wang et al., 2012; Wang N.Y. et al., 2016; Miles et al., 2013; Carstens et al., 2017). As this disease was previously thought to be absent in Mediterranean countries like Spain, Italy, Israel, and Turkey, *P. citricarpa* was listed as an A1 quarantine pest by European Union (Paul et al., 2005; EFSA, 2014). However, a recent survey reported the presence of *P. citricarpa* in Italy, Malta and Portugal (Guarnaccia et al., 2017).

Besides *P. citricarpa*, other species of *Phyllosticta* have been reported to be associated with citrus. *P. citriasiana*, first identified to be a harmful pathogen of pomelos in 2009, was able to cause necrotic spots (tan spots) on fruit similar to those caused by *P. citricarpa* (Wulandari et al., 2009). By performing multi-locus phylogenetic analyses on a large number of *Phyllosticta* species collected in China, Wang et al. (2012) found that *P. citriasiana* was isolated only from pomelos, and *P. citricarpa* was isolated from lemons, mandarins, and oranges, but never from pomelos, indicating that the citrus-associated pathogenic *Phyllosticta* may have a host-related differentiation (Wang et al., 2012). In addition to *P. citriasiana*, many other *Phyllosticta* fungi have also been reported from citrus, such as *P. capitalensis*, *P. citribraziliensis*, *P. citrichinaensis*, *P. paracapitalensis*, and *P. paracitricarpa* (Glienke et al., 2011; Wang et al., 2012; Guarnaccia et al., 2017, 2019). Of them, *P. capitalensis* is the most frequently isolated species. This species has a very wide distribution and it has been isolated as endophytes from dozens of plants as well as pathogens of several plant species (Aa, 1973; Wikee et al., 2013).

As the important causal agent of CBS, *P. citricarpa* has been extensively studied and much information is now available on this pathogen’s population structure, reproduction mode and introduction pathways (Spósito et al., 2011; Wang N.Y. et al., 2016; Carstens et al., 2017). However, little is known about the newly identified pathogen of pomelo tan spot, *P. citriasiana*. In this study, we sequenced the genome of *P. citriasiana*, generating a high-quality reference genome assembly and provide an overview of the genome structure of this important pathogen; we also compared its genome with other two closely related *Phyllosticta* species associated with citrus, i.e., *P. citricarpa* and *P. capitalensis* to provide insight into their evolutionary conservation and diversification.

## Materials and Methods

### Fungal Strain

The *Phyllosticta citriasiana* strain ZJUCC200914 (deposited at China General Microbiological Culture Collection Center with the accession number CGMCC3.14344) was isolated from tan spot infected pomelos collected from Fujian Province, China (Wang et al., 2012). Cultures of strains were maintained on regular solid PDA (potato dextrose agar) or in liquid potato dextrose broth (PDB) at 25°C.

### Genome Assembly and Annotation

The genomic DNA and RNA of *P. citriasiana* were extracted from mycelia grown in PDB as described previously (Wang et al., 2015). The genome was first surveyed through Illumina HiSeq 2500 platform using TruSeq libraries (150 bp paired-end reads, insert size of 350 bp) and then sequenced using the long reads PacBio technology. A total of 1.9 Gb PacBio data, 6.7 Gb pair-end data were generated in the sequencing process, which corresponds to ∼250 fold of sequence depth. To obtain high-quality gene calls, RNA-Seq was conducted with the same sample and 6.0 Gb Illumina paired-end reads were obtained.

To obtain the *P. citriasiana* genome, the PacBio reads were initially assembled using Canu 1.7 (Koren et al., 2017) and error correction was conducted using Pilon version 1.22 with the Illumina reads (Walker et al., 2014). Genome quality assessment was performed through the presence of conserved single-copy fungal genes using BUSCO version 3 (−l Pezizomycotina_odb9) (Simao et al., 2015). RNA-seq data were aligned to the genome using TopHat 2.0.9 with default parameters (Kim et al., 2013). Genome annotation was performed using the BRAKER version 1.0 pipeline combining the RNA-seq-based gene prediction and *ab initio* gene prediction (–fungus, –bam = accepted_hits.bam) (Hoff et al., 2015).

The genomes of *P. citricarpa* (accession number LOEO00000000.1) and *P. capitalensis* (accession number LOEN00000000.1) were downloaded from the NCBI genome database (Wang N.Y. et al., 2016). Gene model predictions of these two genomes were generated with AUGUSTUS 3.1 using the training annotation file of *P. citriasiana* (–genemodel = complete) (Stanke et al., 2008). Repetitive elements were annotated in all assemblies using RepeatMasker version open-4.0.7^1^. For pairwise syntenic analysis of genome structures, the contigs of the paired genomes of *Phyllosticta* species were aligned with the MUMmer 3.23 package (–num, -L 1000) (Delcher et al., 2003). The average nucleotide identity was estimated using the ANI calculator (Rodriguez-R and Konstantinidis, 2016). The statistical reports for genomes were calculated by using in-house Perl scripts.

### Functional Annotation of Genes

To functionally annotate gene models, we assigned protein sequence motifs to protein families (Pfam) and gene ontology (GO) terms using the Pfam and eggNOG databases (Huerta-Cepas et al., 2017; El-Gebali et al., 2018). The GO enrichment in molecular functions was produced with the dcGO database (Fang and Gough, 2013). Protein orthogroups were clustered using the orthoMCL algorithm in combination with an all-versus-all protein BLAST search (*e*-value < 1e-10, identity >50%) (Chen et al., 2006). The carbohydrate-active enzymes were annotated by the web-based dbCAN2 meta server (supported by > = 2 tools) (Zhang et al., 2018). To identify secreted proteins, we use SingalP 4.1 to predict the transmembrane domains (PredHel <2) and we excluded non-extracellular and GPI-anchored proteins by using targetP 1.1 (Emanuelsson et al., 2000) and GPI-SOM (Fankhauser and Mäser, 2005). Fungal secondary metabolite pathways were predicted using the online tool antiSMASH 4.0 (Blin et al., 2017). To estimate the dN/dS ratio, the orthologous proteins between *P. citriasiana* and the other two *Phyllosticta* species were first aligned. After removing the gap in the alignment, the amino acid sequences were replaced by the corresponding coding DNA sequences. The resulting codon alignment was then entered into PAML to calculate the Ka and Ks value with the Yang and Nielsen method (Yang and Nielsen, 2000; Yang, 2007).

## Results and Discussion

### Genome Assembly and General Features

We assembled the genome of *P. citriasiana* using a combination of Illumina and PacBio reads with ∼250 fold of sequence depth. The *de novo* assembly resulted in a genome size of 34.2 Mb assembled in 92 contigs with an N50 of ∼1 Mb. The genomes of *P. citricarpa* and *P. capitalensis* previously sequenced were utilized and annotated in this study (Wang N.Y. et al., 2016). The completeness of these three genome assemblies was estimated by BUSCO (Simao et al., 2015). We found 3095 out of 3156 (98.1%) BUSCO groups were identified in the *P. citriasiana* genome, indicating a high degree of completeness. Although the assembly of *P. citricarpa* and *P. capitalensis* possess a large number of contigs, the BUSCO results showed that they are around 95% completeness, suggesting that these genomes are reliable for the downstream analyses (Table 1).

**TABLE 1:** Genomic features of three *Phyllosticta* species associated with citrus.

**Features**	***P. capitalensis* strain Gm33**	***P. citricarpa* strain Gc12**	***P. citriasiana* strain CGMCC3.14344**
Genome size (Mb)	32.4	31.1	34.2
BUSCOs (%)	94.4	94.5	98.1
Number of contigs	1341	5748	92
N50 (Kb)	20.8	76	968.9
GC content (%)	54.6	53.1	51.4
Protein-coding genes	9983	9083	9202
Gene density (number of genes per Mb)	308	292	269
Mean gene length (bp)	1632	1642	1677
Repeat rate (%)	0.29	0.97	2.19

The overall G + C content of *P. citriasiana* (51.4%) is apparently lower than that of *P. citricarpa* (53.1%) and *P. capitalensis* (54.6%). The percentage of repetitive sequences of *P. citriasiana* is 2.19%, around 2-fold of that of *P. citricarpa* (0.97%) and 7-fold of *P. capitalensis* (0.29%). The actual percentage of repetitive sequences on the genomes of *P. citricarpa* and *P. capitalensis* is expected to be larger as repetitive sequences are the major factor contributing to the fragmented assembly of the genomes. *P. citriasiana* has the lowest gene density but the longest gene length among the three species. The genome of *P. citriasiana* was predicted to have 9202 proteins, which is comparable with that of *P. citricarpa* (9083), but much lower than that (9983) of *P. capitalensis* genome (Table 1).

During preparing this manuscript, we noticed that a paper describing the genomic sequencing of *P. citricarpa* and *P. capitalensis* was published (Rodrigues et al., 2019). However, the general features of their genome sequences are of great difference from ours. For example, the authors predicted ∼15000 proteins for both species while our data only predicted ∼9500 ones. In that study, we found that the *P. citricarpa* genomic assembly consisted of 19,143 contigs with the N50 of 3049 bp and the *P. capitalensis* genomic assembly contains 11,080 contigs with the N50 of 4925 bp (Rodrigues et al., 2019). This means that their genome sequence was very fragmented and the incompleteness of the genome was also confirmed by their BUSCO analysis (Rodrigues et al., 2019). Thus, comparing with their genomic data, we believe that the data in this study is much better and more reliable. In the manuscript review process, a review paper focusing on identification, distribution, genomics, epidemiology and disease management of *Phyllosticta* species was published and provided the genomic data of six species without detailed comparison (Guarnaccia et al., 2019). The genomic data will be of great value for future studies, however, there exist some differences between their genomic features and ours in the respects of assembly length and gene numbers. Taking *P. citriasiana* for example, their genome was 32.7 Mb in length with 11368 predicted genes while ours was 34.2 Mb with only 9202 genes (Guarnaccia et al., 2019). This should be related to the different methods used in the genome assembly and gene prediction. Braker (the software used in this study) tends to predicted less genes than other softwares, such as Maker, but with a reliable accuracy (Hoff et al., 2015; Min et al., 2017).

### Genomic Similarity

**FIGURE 1:**
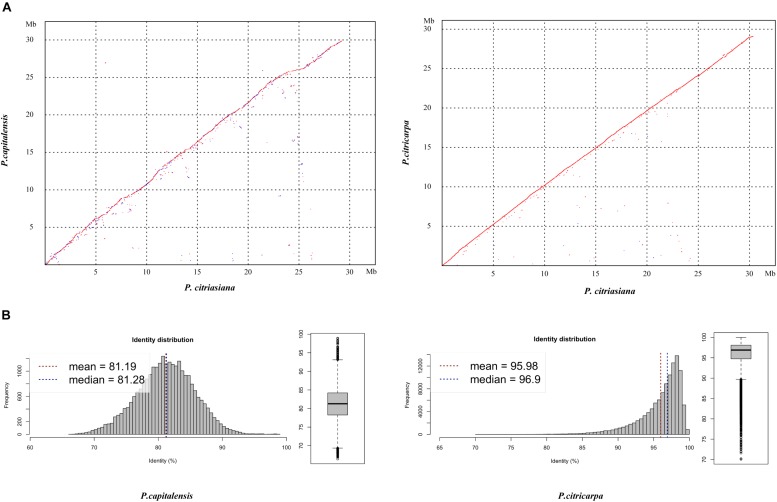
Genomic similarity between *Phyllosticta citriasiana* and the other two *Phyllosticta* species (*P. citricarpa* and *P. capitalensis*) associated with citrus. **(A)** Dotplos showing genome nucleotide alignments of *P. citriasiana* with *P. citricarpa* and *P. capitalensis*. Red diagonals represent alignments in the same direction, whereas blue ones suggest a reverse orientation. **(B)** Distribution of nucleotide identities between *P. citriasiana* and the other two *Phyllosticta* species.

The pairwise comparison analysis based on oriented contigs reveals a high degree of genome-wide macrosynteny between *P. citriasiana* and the other two species (Figure 1A). However, the average nucleotide identity (95.98%) between *P. citriasiana* and *P. citricarpa* is much larger than that (81.19%) between *P. citriasiana* and *P. capitalensis* (Figure 1B), indicating that *P. citriasiana* is much closer to *P. citricarpa* and relatively far from *P. capitalensis*. This result coincides with the fact that *P. capitalensis* is an endophyte of citrus while *P. citriasiana* and *P. citricarpa* are host-specific pathogens (Wang et al., 2012; Guarnaccia et al., 2019).

### Carbohydrate Active Enzymes and Secretomes

The cell wall in plant forms a complex network of different polysaccharides that includes cellulose, hemicellulose, pectin, and lignin. Carbohydrate-active enzymes (CAZymes) play important roles in the breakdown of complex carbohydrates and are responsible for the acquisition of nutrients from the plant for plant-associated fungi (Kubicek et al., 2014). A total of 267 putative CAZyme genes were identified in *P. citriasiana*, which includes 140 Glycoside Hydrolases (GHs), 62 Glycosyl Transferases (GTs), 48 Auxiliary Activities (AAs), 10 Carbohydrate Esterases (CEs), 6 Polysaccharide Lyases (PLs), and 1 Carbohydrate-Binding Modules (CBMs) (Supplementary Tables S1–S4). The types and numbers of CAZymes among different species of *Phyllosticta* are very similar (Supplementary Tables S1–S4). When compared with other species in the Dothideomycetes, *P. citriasiana* appears to contain a less extensive set of CAZymes, for example, *Alternaria alternata* has 373 CAZymes while *Zymoseptoria tritici* contains 324 ones (Goodwin et al., 2011; Ohm et al., 2012; Wang M. et al., 2016). It has been reported that *Phyllosticta* species have a relatively long time to infect citrus fruits and the scab expanded slowly in the fruit peels (Wang et al., 2012; Goulin et al., 2016), however, it is still unclear whether this phenomenon is related to the smaller number of CAZymes in these species.

Pathogens can secrete a series of proteins that are deployed to the host-pathogen interface during infection, and secretome proteins play an important role in pathogenicity (Presti et al., 2015). Approximately 5% of the total proteins (470) of *P. citriasiana* are predicted to be secreted (Supplementary Tables S5–S8). “Hydrolase activity” was the most abundant molecular function of the secretome, other GO terms over-represented among the secreted proteins include “hydrolase activity, acting on glycosyl bonds”, “carboxylic ester hydrolase activity”, “lipase activity”, “exopeptidase activity”, “serine hydrolase activity”, and “hydrolase activity, acting on carbon-nitrogen bonds” (Table 2). The other two *Phyllosticta* species contain a similar number of SPs with *P. citriasiana*, i.e., 465 in *P. citricarpa* and 491 in *P. capitalensis* (Supplementary Tables S5–S8). However, their GO categories showed some differences from that of *P. citriasiana*. The SPs of *P. citricarpa* were not enriched in “exopeptidase activity”, “serine hydrolase activity”, and “hydrolase activity, acting on carbon-nitrogen bonds” but was in “transferase activity, transferring hexosyl groups” (Table 2). The SPs of *P. capitalensis* lacks “exopeptidase activity” and “hydrolase activity, acting on carbon-nitrogen bonds” but contains “phosphatase activity”, “transferase activity, transferring hexosyl groups”, and “UDP-glycosyltransferase activity” (Table 2). These results suggested that the constitution of different *Phyllosticta* secretomes has changed, each species have some preferred enzymes with distinct activities.

**TABLE 2:** Enriched molecular functional categories for secreted proteins of *Phyllosticta* species associated with citrus.

**Species**	**GO id**	**GO term**	**FDR**	**Gene number**
*P. citriasiana*	GO:0016787	Hydrolase activity	2.82E-21	82
	GO:0016798	Hydrolase activity, acting on glycosyl bonds	1.34E-19	29
	GO:0052689	Carboxylic ester hydrolase activity	4.44E-07	12
	GO:0016298	Lipase activity	3.61E-05	10
	GO:0008238	Exopeptidase activity	8.67E-04	8
	GO:0017171	Serine hydrolase activity	6.66E-03	7
	GO:0016810	Hydrolase activity, acting on carbon-nitrogen bonds	9.33E-03	8
*P. citricarpa*	GO:0016787	Hydrolase activity	4.80E-17	76
	GO:0016798	Hydrolase activity, acting on glycosyl bonds	1.57E-20	30
	GO:0052689	Carboxylic ester hydrolase activity	4.56E-06	11
	GO:0016298	Lipase activity	2.10E-04	9
	GO:0016758	Transferase activity, transferring hexosyl groups	2.42E-03	10
*P. capitalensis*	GO:0016787	Hydrolase activity	1.48E-18	100
	GO:0016798	Hydrolase activity, acting on glycosyl bonds	6.07E-15	29
	GO:0052689	Carboxylic ester hydrolase activity	5.68E-08	15
	GO:0016298	Lipase activity	1.76E-04	11
	GO:0016791	Phosphatase activity	3.96E-04	13
	GO:0016758	Transferase activity, transferring hexosyl groups	4.07E-04	14
	GO:0017171	Serine hydrolase activity	1.14E-03	10
	GO:0008238	Exopeptidase activity	1.90E-03	9
	GO:0008194	UDP-glycosyltransferase activity	6.87E-03	8

### Orthologs Groups and Orphan Genes

**FIGURE 2:**
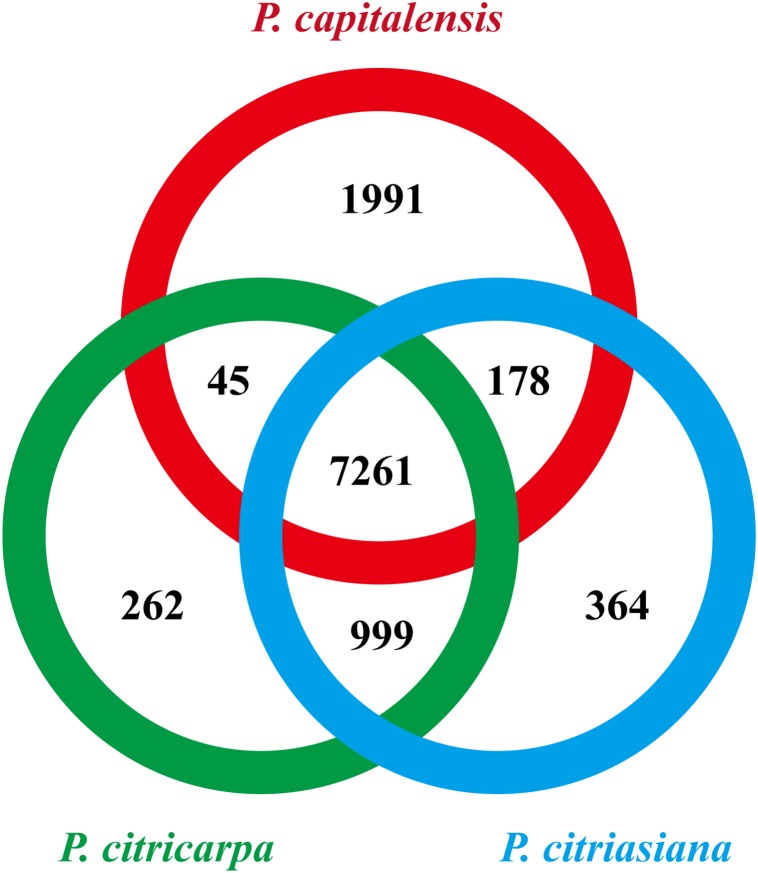
Numbers of orthologous groups that are unique to each isolate, specific to two isolates, and common to all three *Phyllosticta* isolates.

We then searched the conservation and diversification of proteins among different *Phyllosticta* species from the genome-scale. The protein orthology analysis identified 7261 orthologous groups that existed in all the three *Phyllosticta* species, constituting the core gene set of *Phyllosticta* (Figure 2). To find if any protein might be under positive selection, the dN/dS ratios for predicted proteins in a pairwise comparison between *P. citriasiana* and the other two *Phyllosticta* species were calculated. However, all genes show signs of purifying selection (dN/dS < 1). 1991 genes in *P. capitalensis* have no orthologs in the other two species (Figure 2), suggesting that these genes might play roles in constructing the endophytic or pathogenic relationship of *P. capitalensis* with its hosts. *P. citriasiana* and *P. citricarpa* encoded 364 and 262 species-specific proteins, respectively (Figure 2), suggesting that these genes might be related to the host-specific pathogenicity. To know the functions of the genes in those three gene sets, we annotated them using the eggNOG database. However, the results showed that the majority of genes in each group encoded proteins without well-characterized domains and very few sequences can be assigned to the GO terms (Supplementary Tables S9–S12).

We are then curious about if the distribution of CAZymes and secreted proteins might differ among different gene sets. We found that the *P. capitalensis* orphan genes contain only 3 CAZyme genes which encode an AA3 family of glucose oxidase, a GT90 family of xylosyltransferase, and a CE5 family of cutinase while the other two species’ orphan genes contain no CAZyme gene (Supplementary Table S13). However, the case for the secreted proteins is much different. *P. capitalensis*, *P. citricarpa*, and *P. citriasiana* contain 75, 8, and 17 species-specific secreted proteins, respectively (Supplementary Table S13). These results indicate that *Phyllosticta* species have formed lineage-specific sets of orphan genes which might have a potential role in species diversification. Although functions of most orphans are unknown, the secreted proteins (potential effectors) are likely the essential factors of host specialization and they might be good candidates for future functional characterization in distinct *Phyllosticta* species.

### Secondary Metabolite Gene Clusters

**FIGURE 3:**
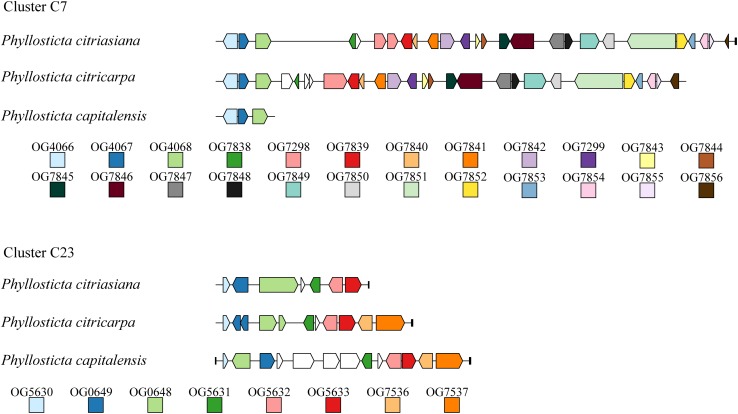
Structural variations of secondary metabolic (SM) gene cluster C7 and C23 among *Phyllosticta* species associated with citrus. For each SM cluster, ortholog among different species are marked with the same color. Genes marked by white lack orthologs in other species. The short black vertical line indicates the end of the contig.

We identified 23 secondary metabolites (SM) biosynthesis clusters in the three *Phyllosticta* species (Supplementary Table S14). These clusters are comprised of 3 NRPS clusters, 5 PKS clusters, 4 terpene clusters, 1 terpene-NRPS cluster, 1 PKS-NRPS cluster, and 9 clusters do not fit into any category (Supplementary Table S14). Of them, cluster C9 contains all *Alternaria solani* genes involved in alternapyrone synthesis, suggesting that these *Phyllosticta* species have the potential to synthesize alternapyrone or its derivatives (Fujii et al., 2005). Most SM clusters (21) are well conserved among the three *Phyllosticta* species while 2 SM clusters of them showed whole cluster gain and loss polymorphisms or gene content polymorphisms. Cluster C7 was present in *P. citricarpa* and *P. citriasiana* but absent from *P. capitalensis*, indicating that this cluster might be lost in *P. capitalensis* or gained in the common ancestor of *P. citricarpa* and *P. citriasiana*. Meanwhile, Cluster C7 in *P. citricarpa* possesses another 3 genes while *P. citriasiana* contains a ∼11 Kb region encoding no proteins, showing gene content polymorphisms (Figure 3). SM cluster C23 showed two gene content polymorphisms. One is that the *P. capitalensis* has an additional four genes between orthologous gene OG0649 and OG0648. The other is *P. citriasiana* lost two genes, of which gene OG7537 encodes the backbone of this cluster (Figure 3). A following tBLASTn analysis against the *P. citriasiana* genome confirmed the loss of these two genes. This gene content polymorphism was most likely generated through a genomic deletion event, rendering the SM gene cluster non-functional.

Secondary metabolites, especial fungal toxins, are believed to be involved in the pathogenicity of many plant pathogenic fungal species and can be described as potential virulence factors. Previously, a handful of secondary metabolites from the citrus pathogen *P. citricarpa* were identified and characterized. Of them, a new dioxolanone, phenguignardic acid butyl ester, showed low phytotoxic activity in citrus leaves and fruits (at a dose of 100 μg) (Savi et al., 2019). However, the involvement of this compound in the formation of citrus black spot disease needs to be further addressed. In this study, we observed the major structural variation of two SM clusters among different *Phyllosticta* species, therefore, distinct corresponding metabolites are expected. However, if they are involved in the host specialization are not known. So, future investigations and elucidations of secondary metabolic mechanisms in *Phyllosticta* species and their functions involved in plant-fungal interactions will be of great significance.

## Conclusion

*Phyllosticta capitalensis*, *P. citricarpa*, and *P. citriasiana* are three very important *Phyllosticta* species associated with citrus (Guarnaccia et al., 2019). *P. capitalensis* is an endophyte fungus of citrus while *P. citricarpa* can cause black spot of citrus (e.g., oranges and mandarins) (Kotzé, 1981; Wang et al., 2012). *P. citriasiana* was identified recently which is the causal agent of the pomelo tan spot (Wulandari et al., 2009; Wang et al., 2012). So far, a systematically comparative genomics analysis among these species is absent. Our results showed that large-scale genome synteny exists and more than 65% of orthologs groups were shared among these species. The similarity between *P. citricarpa* and *P. citriasiana* is more striking with the mean nucleotide identity of ∼96%. Such large scale genome conservation was expected since macrosynteny is often observed among taxa that are phylogenetically closely related (Ohm et al., 2012; Wang M. et al., 2016). Carbohydrate-active enzymes (CAZymes) is thought to be of importance for the breakdown of plant cell wall and are responsible for the acquisition of nutrients from the plant for plant-associated fungi (Kubicek et al., 2014). However, the types and numbers of CAZymes among different species of *Phyllosticta* are very similar. The content of CAZymes seems to be unrelated with host specialization. Although the number of secreted proteins of different *Phyllosticta* species is also very similar, the molecular function of different *Phyllosticta* secretomes has changed, and each species has some preferred enzymes with distinct activities. Effector genes usually evolve rapidly in response to selection pressure exerted by the host, causing the functional diversification in closely related species (Presti et al., 2015). Moreover, sets of orphan genes were detected for each species. The majority of the orphan genes encoded proteins without well-characterized domains, but they contain a certain number of species-specific secreted proteins. Hence, effector proteins should remain the focus of future research. Finally, we observed that 2 SM clusters of *Phyllosticta* showed whole cluster gain and loss polymorphisms or gene content polymorphisms. These types of variation in SM gene clusters have been recorded to be important driving forces for the fungal SM gene cluster divergence (Lind et al., 2017). Therefore, distinct corresponding metabolites are expected.

Deciphering the genetic basis of the transition between closely related pathogenic and endophytic fungi is crucial for a better understanding of the evolutionary history of fungal lifestyles. A previous report identifies genomic signatures indicative of an evolutionary transition from pathogenic *Colletotrichum incanum* to endophytic *C. tofieldiae*, including a narrowed repertoire of secreted effector proteins and expanded secondary metabolism-related proteins (Hacquard et al., 2016). However, these signatures are not observed in our comparative analyses of the three *Phyllosticta* species. An enormous number of orphan genes, a set of preferred secreted proteins and variations in some SM gene clusters are the main features for the endophytic *P. capitalensis* to be distinct from the pathogenetic *P. citricarpa* and *P. citriasiana*. It is commonly known that *P. capitalensis* is an endophyte in a wide range of hosts as well as a weak pathogen of several plant species (Aa, 1973; Wikee et al., 2013), thus, the *P. capitalensis* genome is speculated to include different forms of interaction. Future investigation of the population genomes of *P. capitalensis* might provide more insights into the underlying mechanism.

Taken together, we sequenced the genome of *P. citriasiana*, the causal agent of the pomelo tan spot, generating a high-quality reference genome assembly and provide an overview of the genome structure of this important pathogen. We performed comparative genomics analysis to reveal overall high similarities in sequence identity and gene content among *P. citriasiana*, *P. citricarpa*, and *P. capitalensis*. Our data also highlighted several striking differences in the constitution of secretomes, species-specific genes, and secondary metabolite gene clusters. However, it is yet to be determined how a *Phyllosticta* species emerged as a pathogen of alternate hosts. These data would be valuable in the future investigation of the driving forces of fungal host switch, in population genomic studies for identification of haplotypes and alleles, and in identifying effectors that may function in infection of citrus plants.

## Data Availability Statement

The assembled *Phyllosticta citriasiana* genome has been deposited in GenBank under the accession number QOCM00000000. All the annotation data generated in this study have been deposited on the figshare repository at https://doi.org/10.6084/m9.figshare.9178061.

## Author Contributions

MW and HL conceived the study. All authors analyzed the data and reviewed the manuscript. MW wrote the manuscript.

## Conflict of Interest

The authors declare that the research was conducted in the absence of any commercial or financial relationships that could be construed as a potential conflict of interest.

## Footnotes

1 http://www.repeatmasker.org
